# Transplant Oncology: An Evolving Field in Cancer Care

**DOI:** 10.3390/cancers13194911

**Published:** 2021-09-29

**Authors:** Maen Abdelrahim, Abdullah Esmail, Ala Abudayyeh, Naoka Murakami, Ashish Saharia, Robert McMillan, David Victor, Sudha Kodali, Akshay Shetty, Joy V. Nolte Fong, Linda W. Moore, Kirk Heyne, A. Osama Gaber, Rafik Mark Ghobrial

**Affiliations:** 1 Section of GI Oncology, Department of Medical Oncology, Houston Methodist Cancer Center, Houston, TX 77030, USA; AEsmail@houstonmethodist.org (A.E.); Heyne@houstonmethodist.org (K.H.); 2 Cockrell Center of Advanced Therapeutics Phase I Program, Houston Methodist Research Institute, Houston, TX 77030, USA; 3 Department of Medicine, Weill Cornell Medical College, New York, NY 10065, USA; ASaharia@houstonmethodist.org (A.S.); rrmcmillan@houstonmethodist.org (R.M.); dwvictor@houstonmethodist.org (D.V.); SKodali@houstonmethodist.org (S.K.); ashetty@houstonmethodist.org (A.S.); AOGaber@houstonmethodist.org (A.O.G.); RMGhobrial@houstonmethodist.org (R.M.G.); 4 Section of Nephrology, Division of Internal Medicine, The University of Texas MD Anderson Cancer Center, Houston, TX 77030, USA; AAbudayyeh@mdanderson.org; 5 Division of Renal Medicine, Brigham and Women’s Hospital, Harvard Medical School, Boston, MA 02115, USA; nmurakami1@bwh.harvard.edu; 6 Sherrie and Alan Conover Center for Liver Disease and Transplantation, JC Walter Jr. Center for Transplantation, Houston Methodist Hospital, Houston, TX 77030, USA; jvnoltefong@houstonmethodist.org (J.V.N.F.); LWMoore@houstonmethodist.org (L.W.M.)

**Keywords:** transplant oncology, liver transplantation, cholangiocarcinoma, neuroendocrine tumor, liver metastases, hepatocellular carcinoma, circulating tumor DNA, colorectal cancers, immunotherapy

## Abstract

**Simple Summary:**

Transplant oncology is an emerging concept of cancer treatment with a promising prospective outcome. The application of oncology, transplant medicine, and surgery to improve patients’ survival and quality of life is the core of transplant oncology. This review illustrates the concept and history of transplant oncology as an evolving field for the management of hepatocellular carcinoma, intrahepatic biliary cancer, and liver-only metastasis of non-hepatobiliary carcinoma. The utility of immunotherapy in the transplant setting is discussed as well as the feasibility of using circulating tumor DNA for surveillance post-transplantation. As transplant oncology continues to evolve as a promising field in cancer management, it is expected that there will be improved outcomes and expansion of transplant eligibility through the consolidation of multidisciplinary and collaborative efforts. Liver transplantation is increasingly associated with improved survival outcomes in patients with liver malignancies. Eligibility criteria for liver transplantation has expanded over the years to include more patients with cancer. In addition, immunotherapy and ctDNA are two emerging concepts that are highly applicable to transplant oncology treatment. Immunotherapy may be used as neoadjuvant “bridging” therapy pre- liver transplantation and possibly in the palliative setting post-transplantation. Liquid biopsy to assess ctDNA post-transplantation can potentially be used as a biomarker to detect minimal residual disease and disease recurrence.

**Abstract:**

Transplant oncology is an emerging concept of cancer treatment with a promising prospective outcome. The application of oncology, transplant medicine, and surgery to improve patients’ survival and quality of life is the core of transplant oncology. Hepatobiliary malignancies have been treated by liver transplantation (LT) with significant improved outcome. In addition, as the liver is the most common site of metastasis for colorectal cancer (CRC), patients with CRC who have stable unresectable liver metastases are good candidates for LT, and initial studies have shown improved survival compared to palliative systemic therapy. The indications of LT for hepatobiliary malignancies have been slowly expanded over the years in a stepwise manner; however, they have only been shown to improve patient survival in the setting of limited systemic therapy options. This review illustrates the concept and history of transplant oncology as an evolving field for the management of hepatocellular carcinoma, intrahepatic biliary cancer, and liver-only metastasis of non-hepatobiliary carcinoma. The utility of immunotherapy in the transplant setting is discussed as well as the feasibility of using circulating tumor DNA for surveillance post-transplantation.

## 1. Introduction

Transplant oncology is an evolving concept of cancer treatment with a promising prospective outcome. The application of oncology, transplant medicine, and surgery to improve patients’ survival and quality of life is the core of transplant oncology. Hepatocellular carcinoma (HCC), the most common liver malignancy [1], has been treated by transplantation with excellent outcomes. Hepatobiliary cancer management and research can benefit from integrating the principles of transplant oncology in an attempt to use current knowledge to guide future opportunities. Liver transplantation (LT) for selected liver malignancies is the only solid organ transplant with noticeable efficacy in curing cancer. Transplant oncology can potentially contribute to the treatment and research of hepatobiliary malignancies in four ways: ref. [1] exploring a new concept of cancer treatment that includes LT, ref. [2] connecting tumor and transplant immunology and pursuing translational research in self and non-self-recognition, ref. [3] developing innovative clinical and experimental standards for accessing and exploiting the explanted liver, and ref. [4] using a multidisciplinary approach to hepatobiliary oncology to overcome the limitations of current surgical techniques [2]. 

## 2. Concept and History of Transplant Oncology

The main goal of transplant oncology is to improve the survival outcomes and quality of life for cancer patients by removing the cancerous organ completely and replacing it with a healthy organ. In addition, to expand the boundaries of treatment and research for hepatobiliary cancers, the concept of transplant oncology encompasses multiple disciplines of transplantation medicine and oncology. A portion of this concept is constituted by LT for hepatobiliary malignancies. On February 7, 2019, the international Liver Transplantation Society (ILTS) held a conference in Rotterdam, Netherlands on transplant oncology [5]. This consensus conference is probably considered as the inaugural moment of transplant oncology disciplines resulting in the release of the first consensus guidelines.

**Figure 1:**
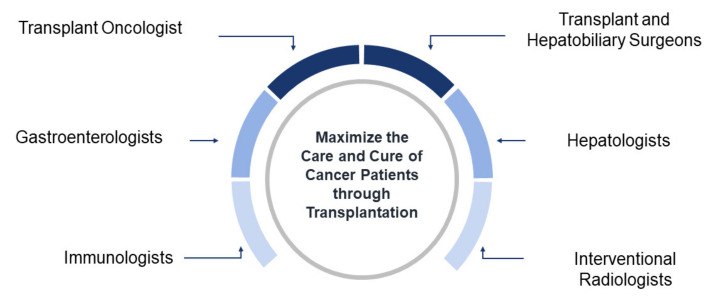
Concept map of transplant oncology. Multidisciplinary collaborative approach to maximize the care of cancer patients through transplantation.

Many factors have contributed to help transplant oncology evolve from a concept toward an anticancer strategy, such as initiating studies in genomics and cancer immunogenomics based on new insights in liver cancer. In addition, the adoption of surgical transplantation techniques in oncology have improved conventional resection and bridged the gap between tumor and transplant immunology. The continuing collaboration between the relevant subspecialties, including transplant oncologists, hepatologists, gastroenterologists, transplant hepatobiliary surgeons, interventional radiologists, and immunologists, will improve treatments and cure rates for hepatobiliary and other cancer patients (Figure 1) [3]. 

### 2.1. Liver Transplantation for Hepatocellular Carcinoma

Unlike other solid organ transplantation, where history of active cancer tends to be considered as contraindication for transplantation, liver transplantation is one of the special occasions that can be used as a curative approach for malignancy. HCC has curative treatment options that include surgical resection in patients with well-compensated liver function and radiofrequency ablation in those with small tumors. However, in 90% of patients, HCC occurs in the setting of cirrhosis, where optimal management remains LT, with 5-year survival rates of approximately 80% [4]. Thus, LT offers an optimal treatment option in a specific subgroup of patients with HCC. The guidelines have been clinically modified to help select HCC patients for both deceased and living donor LT. The eligibility criteria can consider many factors besides the tumor size and number, such as tumor biology (including alpha-fetoprotein [AFP] level), transplant benefit (i.e., the likelihood of survival on the waitlist and after LT), availability of donor organs, and composition of the waitlist. These modifications in eligibility criteria at some transplant centers were done in response to the increasing incidence of HCC and the excellent survival outcomes of LT for HCC [4].

#### 2.1.1. Milan Criteria

Substantial efforts were made in Milan, Italy by Mazzaferro et al. [6], who established a model for patients with unresectable HCC to be treated by LT. This study established the Milan criteria: the tumor diameter of a single lesion less than or equal to 5 cm, or for multiple lesions, no more than three tumors, each less than or equal to 3 cm, without vascular invasion or extrahepatic metastases. Furthermore, patients who meet the criteria must have their HCC diagnosis confirmed by either tissue biopsy or serum AFP assay. The study’s results showed that LT is an effective treatment for patients who have cirrhosis and small, unresectable HCCs. These outcomes made the Milan criteria the primary reference for determining which HCC patients will benefit from LT and paved the way for others to invest clinical efforts in this new field.

#### 2.1.2. Beyond Milan Criteria

**Figure 2:**
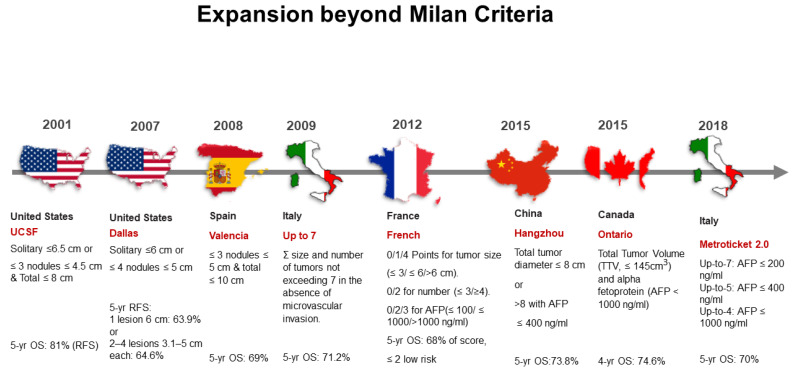
Historical expansions of the Milan criteria for liver transplantation in patients with hepatocellular carcinoma. yr: year; OS, overall survival; RFS, relapse-free survival; ∑, summation; AFP, alpha-fetoprotein; TTV, total tumor volume; ml, milliliters; cm, centimeters; ng, nanograms.

Since 1996, the Milan criteria yielded good outcomes for post-transplant survival recurrence-free survival, yet restricted LT mostly to patients with only small tumors. This encouraged research institutions and hospitals to make efforts to expand the Milan criteria to increase the number of HCC patients who could benefit from LT with favorable prognosis. As a result, the Milan criteria has been modified by many transplant societies to determine whether other patients with HCC can be eligible for LT with an acceptable survival rate of 5 years after transplantation (Figure 2). These efforts expanded the Milan criteria, which depend on the strict tumor size and number of nodules to determine an HCC patient’s eligibility for LT, to depend on overall tumor size and number of nodules as well as different tumor markers such as AFP [7]. This expansion of the criteria has polarized into the classifications of University of California San Francisco (UCSF) and beyond UCSF [8]. Indications of LT for HCC are evolving, and the so-called expanded criteria remains debatable. Locoregional therapies are being used to downstage HCC from beyond to within the Milan criteria. For example, Mazzaferro et al. [9], investigated the efficacy of liver transplantation after successful hepatocellular carcinoma downstaging.

A total of 45 patients with HCC were randomly assigned to the LT group or to the control group after being downstaged. At data cutoff and median follow-up of 71 months, 5-year tumor event-free survival was 76.8% in the transplantation group versus 18.3% in the control group. Five-year overall survival was reported at 77.5%. Authors concluded that after effective and sustained downstaging of eligible hepatocellular carcinomas beyond the Milan criteria, liver transplantation improved tumor event-free survival and overall survival compared with non-transplantation therapies. Post-downstaging tumor response could contribute to the expansion of HCC transplantation criteria.

##### A. UCSF Criteria

UCSF in the Unites States took the first step to expand the Milan criteria. UCSF researchers evaluated the Milan criteria and suggested that the criteria could be modestly expanded, so that UCSF criteria could be used as eligibility guidelines for HCC patients who did not meet the Milan criteria [10]. The UCSF criteria established LT for HCC patients with a single lesion less than or equal to 6.5 cm in diameter or three or fewer lesions less than or equal to 4.5 cm each if the total tumor diameter is less than or equal to 8 cm. The Milan criteria expansion by UCSF showed benefit in an additional 5% to 20% of patients with HCC who would have been excluded from orthotopic LT under the Milan criteria. In addition, with UCSF criteria, a 5-year overall survival rate was 72.4% compared with the Milan criteria’s 4-year overall survival rate of 85% [11].

##### B. Beyond UCSF Criteria

The good survival outcomes from the UCSF criteria encouraged transplant societies around the world to make efforts to maximize the number of patients with unresectable HCC who can benefit from transplant oncology. Numerous studies have expanded the UCSF criteria with great success (Figure 2) [12,13]. For example, criteria from Valencia, Spain included HCC patients with three or fewer nodules, each 5 cm or smaller in diameter, with a total tumor diameter less than or equal to 10 cm [14]. These criteria yielded a 5-year overall survival rate of 69%. Criteria from Hangzhou, China, included HCC patients with total tumor diameter less than or equal to 8 cm or patients with total tumor diameter greater than 8 cm whose AFP level is less than or equal to 400 ng/mL [15]. These criteria yielded a 5-year overall survival rate of 71% [16]. The French criteria featured a point scale in which 2 points or fewer indicates low risk [17]. For tumor size, 0, 1, and 4 points are assigned for lesion diameters less than or equal to 3 cm, greater than 3 but less than or equal to 6 cm, and greater than 6 cm, respectively. For the number of lesions, 0 points are given for three or fewer nodules, while 2 points are given for four or more. For AFP level, 0, 2, and 3 points are given for levels less than or equal to 100 ng/mL, more than 100 ng/mL but less than or equal to 1000 ng/mL, and more than 1000 ng/mL, respectively. These criteria yielded a 5-year overall survival rate of 68% [18]. For the Ontario criteria, patients were eligible based on total tumor volume (TTV) measured less than 145 cm^3^ and AFP is less than 1000 ng/mL. [13]. A recently developed selection system, known as Metroticket 2.0 Model, takes into account tumor size, number, and actual AFP value. Based on this system, patients with HCC have a 70% chance of HCC-specific 5-year survival after transplantation, their level of AFP should be less than 200 ng/mL, and the sum of number and size of tumors (in centimeters) should not exceed 7; if the level of AFP is 200–400 ng/mL, the sum of the number and size of tumors should be less than 5; if their level of AFP is from 400 to 1000 ng/mL, the sum of the number and size of tumors should be less than 4 [12]. In general, various beyond UCSF criteria yielded a 5-year overall survival rate ranging in average from 63% to 81%, which is believed to be an acceptable improvement of survival outcomes compared to no LT. 

### 2.2. Liver Transplant for Non-Hepatocellular Carcinoma Tumors

#### 2.2.1. Cholangiocarcinoma

##### A. Hilar Cholangiocarcinoma

Historically, treatment options for this aggressive cancer have been limited. Resection is the standard of care; however, 5-year survival is only 20–40% [19,20,21,22,23,24,25,26,27]. In addition, many patients present with unresectable disease either due to underlying parenchymal liver disease (primary sclerosing cholangitis) or involvement of bilateral hilar structures [28]. Unresectable hilar cholangiocarcinoma patients have a good chance of successful treatment by LT. Neoadjuvant chemoradiation therapy, when applied to unresectable hilar cholangiocarcinoma patients who undergo LT, has shown an excellent 5-year recurrence-free survival rate of 65% [28]. Many United States transplant centers use the Mayo Clinic protocol of chemoradiation followed by LT to treat unresectable hilar cholangiocarcinoma. In the Mayo protocol, selected patients with unresectable cholangiocarcinoma without intrahepatic or extrahepatic metastases are treated with irradiation plus bolus fluorouracil (5-FU), followed by brachytherapy with iridium and concomitant protracted venous infusion of 5-FU. This is followed by maintenance chemotherapy (i.e., oral capecitabine ambulatory infusion 5-FU) until liver transplantation. Metastatic disease was excluded in this protocol after radiation received by an exploratory laparotomy procedure [29]. This approach has provided excellent outcomes with survival rates of 60.4%. However, the resectability vs. unresectability criteria remain controversial because outcomes from other studies outside the United States have provided modest outcomes [30]. On the other hand, information on the number of centers in the U.S. that are actively transplanting these patients, type of neoadjuvant therapy used, and outcomes of LT achieved has been reported. Murad et al. showed that patients with perihilar cholangiocarcinoma who were treated with neoadjuvant therapy followed by LT at 12 U.S. centers had a recurrence-free survival rate of 65% after 5 years, suggesting this therapy is highly effective [28]. 

##### B. Intrahepatic Cholangiocarcinoma

Patients with intrahepatic cholangiocarcinoma (IHCCA) are often not eligible for LT because of poor outcomes reported from many transplant institutions. These studies, up to the year of 2000, revealed overall survival rates up to 40% at 3 years and 20% at 5 years after LT [31,32,33,34,35]. Over the last 20 years, the outcomes have improved using the approach of a prognostic scoring system developed by researchers at the University of California, Los Angeles [36,37]. This approach recommends the use of neoadjuvant/adjuvant chemotherapy, such as fluorouracil-or capecitabine-based regimens in combination with oxaliplatin, leucovorin calcium, and gemcitabine hydrochloride. In addition, this approach recommends evaluating tumor biology by obtaining tissue biopsy prior to neoadjuvant therapy initiation [38]. The scoring system considers seven clinicopathological risk factors: lack of neoadjuvant or adjuvant treatment, perineural invasion, infiltrative subtype, history of primary sclerosing cholangitis, multifocal tumor, hilar cholangiocarcinoma, and lymph vascular invasion. This scoring system classifies patients as having low, intermediate, and high risk of recurrence to select candidates for LT [36]. Patients in the low-risk group had a 78% 5-year recurrence-free survival rate, compared to 19% for the intermediate-risk group and 0% for the high-risk group [36]. 

Houston Methodist J.C. Walter Jr. Liver Transplant Center and MD Anderson Cancer Center were the first multi-center collaboration sites that reported a prospective case series of protocolized neoadjuvant chemotherapy followed by liver transplantation for patients with IHCCA [39]. This series used no specific tumor size cut-off, and the median cumulative tumor diameter was 14.2 cm. In this series, if tumor radiographic stability was maintained for more than 6 months, patients were evaluated and listed for transplant. The six patients reported on had a 5-year overall survival (OS) of 83.3%, with a 50% recurrence-free survival [40]. More recently, the International Liver Cancer Association recommended prospective clinical trials of neoadjuvant chemotherapy plus LT to treat patients with IHCCA [41]. 

The presence of IHCCA in a cirrhotic liver was a contraindication for liver transplantation in most centers worldwide. However, recent investigations have shown that “very early” IHCCA may have acceptable results after liver transplantation. For example, Sapisochin et al. [42] have reported that in patients with "advanced" disease (single tumor >2 cm or multifocal disease) the 1-year, 3-year, and 5-year actuarial survival rates after LT were 79%, 50%, and 45%, respectively. These findings suggest that patients with cirrhosis and very early IHCCA may become candidates for liver transplantation. More prospective multicenter clinical trials are needed to further confirm these results.

#### 2.2.2. Hepatoblastoma

Hepatoblastoma (HBL) and HCC are the most common primary hepatic malignant neoplasms in childhood. LT combined with chemotherapy is an excellent treatment that provides long-term disease-free survival in children diagnosed with advanced HBL and HCC. Pham TA et al. [43] have shown that pretreatment extent of disease (PRETEXT) stage IV tumors are strongly associated with tumor recurrence and death, whereas metastatic disease in HBL is treatable and is a relative but not absolute contraindication to transplant. Furthermore, more time spent on the transplant waiting list is associated with an increased risk for recurrence in HBL. Hepatocellular carcinoma in children behaves differently than in adults because transplant for lesions well outside the Milan and UCSF criteria results in excellent long-term survival. More investigation is needed to further establish the role of transplant in children with HCC.

## 3. Liver Metastases

### 3.1. Neuroendocrine Tumor Liver Metastases

Recently published data revealed that 5-year OS ranged from 50% to 70% for patients with locally advanced, unresectable neuroendocrine tumor liver metastases (NETLM) who underwent LT; these patients had a 30% to 60% recurrence rate in 5 years [44]. In 2007, Mazzaferro et al. established criteria to select appropriate NETLM patients for LT with curative intent when liver resection or debulking would have limited benefit [45]. These criteria included patients whose primary tumor was a low-grade NET drained by the portal system, had hepatic involvement of at least 50%, showed a response to treatment or had stable disease for at least 6 months, and were of age 55 years or younger. The Milan criteria for NETLM yielded 97% 5-year survival rates and 89% 10-year survival rates in 42 selected patients who underwent LT between 1995 and 2010 [46]. These outcomes led the United Network for Organ Sharing in the United States to adopt the Milan criteria and release guidelines for including patients with unresectable NETLM on transplant lists. Likewise, the European Neuroendocrine Tumor Society Consensus Guidelines and the National Comprehensive Cancer Network Guidelines have released criteria for including patients with unresectable NETLM on lists to potentially receive LT [47,48]. 

### 3.2. Colorectal Cancer

**Figure 3:**
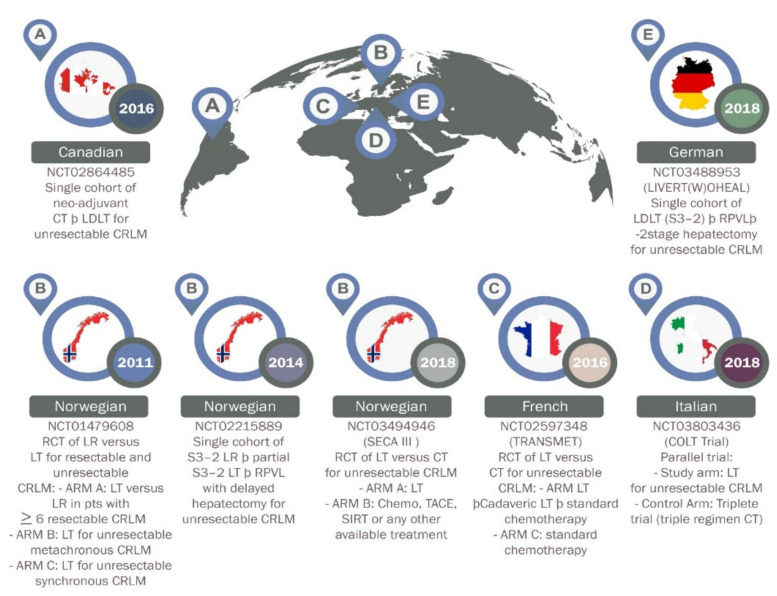
Current prospective trials on liver transplantation for colorectal liver metastases. NCT, National Clinical Trial; CT, chemotherapy; LDLT, living donor liver transplantation; CRLM, colorectal Liver Metastases; LT, liver transplantation; TACE, transarterial chemoembolization; S, stage; SIRT, selective Internal Radiation Therapy; RCT, randomized controlled trial; RPVL, right portal vein ligation; A-E, name of countries as flagged off.

The liver is the most common site of metastasis for colorectal cancer (CRC), the third most common cancer worldwide [49,50]. CRC patients with liver oligometastases are usually treated with curative hepatectomy with good survival outcome. However, patients with CRC who have unresectable liver metastases are usually treated with palliative therapies associated with a relative 5-year survival rate of only about 14% [51]. LT was suggested as a better alternative option for those patients with liver-only, unresectable and stable CRC metastasis. Unfortunately, the initial experience of LT in CRC patients with unresectable liver metastases recorded poor outcomes, with a 5-year overall survival rate lower than 20% [48]. These poor outcomes are believed to be due to the absence of good selection criteria and lack of appropriate neoadjuvant and adjuvant therapies. In recent years, clinical oncology experts have adjusted these standards to be more effective and furthermore, surgical techniques are believed to have improved. As a result, more studies of LT in CRC patients with unresectable liver metastases has been conducted (Figure 3). 

Initially, in the SECA-I trial, Dueland et al. reported on 21 patients with nonresectable liver-only CRC metastasis [52]. This study aimed to compare the disease-free survival (DFS) and OS of this study with progression-free survival (PFS) and OS of the NORDIC VII. The NORDIC VII study was a first-line chemotherapy study with a similar cohort of CRC patients with liver-only disease [53]. While the DFS and PFS in both groups were similar in the range of 8 to 10 months, there was a large difference in OS reported. The 5-year OS rate was 56% in patients post liver transplantation compared with 9% in patients who were only on first-line chemotherapy. The large difference in OS, despite similar PFS, is believed to be due to differences in the metastatic patterns at relapse or progression. Relapse in the liver transplantation group was usually detected as small, slowly growing lung metastases, while larger progression of nonresectable liver metastases was observed in the chemotherapy-only group [52]. 

The SECA-1 trial reported that factors associated with decreased survival included pretransplant maximal tumor diameter exceeding 5.5 cm, level of carcinoembryonic antigen (CEA) before LT > 80 mg/L, failing response on chemotherapy, and short interval from resection of the primary to LT.

In the secondary trial, SECA-II, a total of 15 CRC patients with nonresectable liver-only metastases were evaluated [54]. Patients were required to have at least a 10% response to chemotherapy and more than one year from diagnosis to liver transplant. This study aimed to investigate whether more strict selection criteria can be used to obtain a better OS after LT for CRC and if it was comparable to OS seen for standard indications for LT. The SECA-II study showed a median follow-up of 36 months and a 5-year OS of 83%. Disease-free survival at 1, 2, and 3 years were 53%, 44%, and 35%, respectively. Time from primary surgery to LT, age, plasma CEA value, number of liver lesions, size of largest lesions, Fong Clinical Risk Score (FCRS), Oslo Score, and PET values was compared between the SECA-I and SECA-II trials. Interestingly, none of the patients in the SECA-II study had progressive disease on chemotherapy or CEA levels above 80 mg/L at the time of LT, which might represent good selection factors for future studies. It is believed that LT provides the longest OS reported in patients with colorectal cancer and nonresectable liver metastases. Improved selection criteria can give patients with nonresectable CRC liver metastases a 5-year overall survival that is comparable to other standard indications for LT.

## 4. Emerging Concepts in Transplant Oncology

### 4.1. Immune Therapy in the Peri-Transplant Period

#### 4.1.1. Pretransplant Bridging Therapy

Despite the success of LT in treating HCC, only a small proportion of patients meet the standard Milan criteria to receive a LT due to advanced-stage disease and/or large tumor size preventing and delaying organ allocation. Neoadjuvant “bridging” therapies can effectively downstage disease or delay tumor progression for patients on the LT waiting list [55]. Immune checkpoint inhibitors (ICPIs), such as pembrolizumab and nivolumab, and combinations such as atezolizumab plus bevacizumab (VEGF inhibitor) and nivolumab plus ipilimumab, have achieved deep and long-lasting responses against unresectable HCC. Despite the broad range of adverse events from ICPIs, studies emphasized that they can be tolerated well, with only 15% of patients with unresectable HCC experiencing adverse events requiring treatment discontinuation. However, caution was advised in transplant candidates and recipients by reports of rejection and graft loss, possibly due to dysregulated immune activation [56]. The use of ICPIs as neoadjuvant therapy before surgical resection is a rapidly evolving field. On the other hand, safety and the clinical outcomes of patients receiving immunotherapy as bridging therapy to transplantation remains unknown and it is believed to be an area of future exploration. 

Interestingly, in a recent report [57], nine patients with HCC were transplanted at a single center after receiving ICPI nivolumab as pretransplant bridging therapy. In this report, nivolumab was given at a dose of 240 mg every 2 weeks. It is worth mentioning that 89% of patients received their last dose of nivolumab within 4 weeks of transplant. Immunosuppression used in these patients was methylprednisolone tapered to prednisone over 2 weeks, mycophenolate mofetil, and tacrolimus at a maintenance level of 10–12 ng/mL. Interestingly, at a median follow-up of 16 months post-transplantation, no severe allograft rejections/losses were reported. One patient developed mild acute rejection, due to low tacrolimus levels, which was resolved soon after drug level correction. In addition, during the same median follow-up, no tumor recurrences or deaths were reported. In the explant liver, about one third of patients had near complete (>90%) tumor necrosis [57]. This report opened the door to the possibility of using ICPI as bridging therapy to curative LT. Further investigations of these agents in the pretransplant setting are needed to evaluate the safety and efficacy and to clearly understand the optimal approach for ICPIs to be use in patients waiting for LT. 

#### 4.1.2. Post-Transplant Palliative Therapy

Although immune therapy post-transplant was typically thought to be contraindicated in solid organ transplant recipients due to the risk of allograft rejection, recent reports have shown that LT recipients may be treated with ICPIs in the appropriate setting. Recent reports of LT recipients who were treated with ICPIs showed allograft preservation in nearly two-thirds of the patients [58,59]. These reports evaluated 48 organ transplant patients who received ICPI for advanced cancer. Of these patients, in an Evolution Field of Treating Cancer by Transplantation, 19 cases were liver transplant recipients. The disease control rate was reported at 21% and graft rejection was seen in 37% of liver transplant patients.

In another study, Munker and DeToni reviewed reports on 14 known cases of liver transplant recipients who underwent treatment with ICPI [60]. Factors that might affect susceptibility to organ rejection included the choice of the agent of immunosuppression, PDL-1 status in liver graft biopsies, and the time of treatment initiation. In this report, liver graft rejection was only reported in 4 of 14 reported cases (28%). The median time to rejection occurred within three weeks after the initiation of immune therapy. Per this study, OS was available in 12 cases with a median value of 1.2 months. Interestingly, in 4 patients showing a response to treatment, survival ranged between 4 and 18 months [60].

Furthermore, Rammohan et al. [59], have reported a case of HCC that was initially treated by living donor liver transplantation but progressed to HCC occurrence in the lung 3 years after LT. The patient responded dramatically to ICPI, pembrolizumab, after treatment failure of sorafenib. Pembrolizumab was administered at 200 mg once per 21 days along with sorafenib. Ten months after starting ICPI, the patient remained well and continued on pembrolizumab and sorafenib with no radiological evidence of tumor or graft rejection/dysfunction [59]. In the same direction, De Bruyn et al. have reported on 19 liver transplant patients who were treated with ICPIs for advanced cancers. In this study, 21% of the patients showed disease control and less than 38% of them reported graft rejection leading to the conclusion that liver transplant recipients can be treated with ICPIs [58]. In another retrospective study, Abdel-Wahab et al. [61], reported on 39 patients with allograft transplantation, showing that in the 28% of patients (11 out of 39) with liver transplantation, the median time of ICPIs initiation (both of anti-CTLA-4 and anti-PD-1 therapy) was nine years post transplantation. Allograft rejection occurred only in 41% of all patients enrolled and in the hepatic patients, 4 patients experienced allograft rejection out of 11. 

Data from these reports are insufficient to draw the conclusion that one ICPI or immunosuppressant agent is safer than others. However, it was suggested that liver biopsies of liver allografts should be taken routinely before treatment initiation in LT recipients pre-treatment with steroids should be tried in the absence of contraindications, and immunosuppression should be progressively tapered under close surveillance. Currently, using ICPIs as a therapeutic option, post-LT is still under investigation. The relationship between graft rejection and tumor response is still unclear because there is an insufficient number of cases or studies to evaluate. There are also limited predictive biomarkers to adapt immunotherapy for HCC patients in the setting of post LT [62]. On the other hand, the utility of immunotherapy as bridging therapy to LT has shown more acceptance in the transplant oncology community. In the future, more prospective data are needed to support its safety and efficacy.

### 4.2. Utility of Circulating Tumor DNA (ctDNA) for Cancer Minimal Residual Disease (MRD) Evaluation and Surveillance

**Figure 4:**
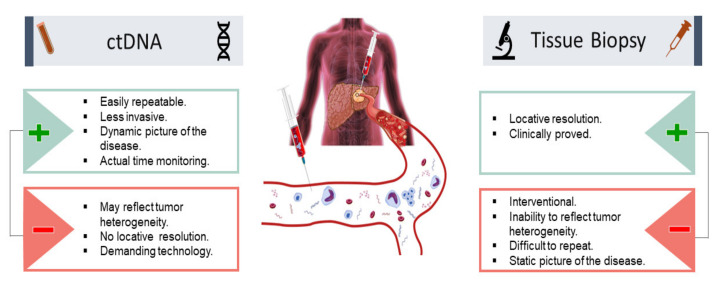
Utility of ctDNA in HCC for minimal residual disease evaluation and surveillance: pros and cons in comparison with tissue biopsy. (+) advantages (−) disadvantages.

Many strategies have been established to survey minimal residual disease (MRD) in HCC patients, such as radiological imaging and tissue biopsy, but many of the strategies have modest sensitivity [63]. Recently, liquid biopsy to assess ctDNA shows promising performance in MRD surveillance for primary liver malignancies (Figure 4) [64]. The ctDNA biopsy measures and analyzes molecular fragments derived from HCC and excreted into the bloodstream. The ctDNA biopsy offers a noninvasive approach that potentially resolves the limited access to HCC tissue samples obtained by tissue biopsy. In addition, the ctDNA biopsy reveals a dynamic picture of HCC, is easily repeatable when needed, and provides real-time surveillance for minimal residual disease in HCC patients. Studies have demonstrated the usefulness of ctDNA in MRD surveillance in HCC [65,66,67]. For example, Kasi et al. analyzed 200 plasma samples from a total of 90 hepatobiliary patients, and of those patients 27 had HCC [68]. It was reported that ctDNA detection was significantly associated with the stage of disease. In addition, serial time point analysis was performed on a subset of patients (*n* = 56) that had 2–7 time points available. Correlations between ctDNA levels and clinical response were presented [66,68,69]. Furthermore, many studies in HCC patients treated with LT demonstrate the clinical usefulness of ctDNA biopsy for detection of tumor progression, MRD surveillance, and early prediction of recurrence (Table 1). 

## 5. Conclusions

As transplant oncology continues to evolve as a promising field in cancer management, it is expected that there will be improved outcomes and expansion of transplant eligibility through the consolidation of multidisciplinary and collaborative efforts. LT is increasingly associated with improved survival outcomes in patients with liver malignancies. Eligibility criteria for LT has expanded over the years to include more patients with cancer. In addition, immunotherapy and ctDNA are two emerging concepts that are highly applicable to transplant oncology treatment. Immunotherapy may be used as neoadjuvant “bridging” therapy pre-LT and possibly in the palliative setting post-transplantation. Liquid biopsy to assess ctDNA post-transplantation can potentially be used as a biomarker to detect minimal residual disease and disease recurrence. 

## Figures and Tables

**Table 1:** Representative circulating tumor DNA studies in HCC.

Study	No. of HCC Patients	Note	Biomarkers	Outcomes
[67]	46	Treatment: Transplant Resection	ctDNA	Detection of ctDNA was associated with increased recurrence
[65]	34	Treatment: TACE Resection RFA	ctDNA	ctDNA can detect minimal residual disease and predict survival
[70]	41	10 controls	TERT, TP53, and CTNNB1	Detection of ctDNA predicted shorter recurrence-free survival
[71]	10	Treatment: TACE Resection RFA	Methylation of GSTP1 and RASSF1A or TP53 mutation	Detecting ctDNA in urine was feasible and predicted

HCC, hepatocellular carcinoma; RFA, radiofrequency ablation; TACE, transarterial chemoembolization.
